# Microbial Fermented Liquid Supplementation Improves Nutrient Digestibility, Feed Intake, and Milk Production in Lactating Dairy Cows Fed Total Mixed Ration

**DOI:** 10.3390/ani13050933

**Published:** 2023-03-04

**Authors:** Sineenart Polyorach, Rutsamee Nampukdee, Metha Wanapat, Sungchhang Kang, Anusorn Cherdthong, Onanong Poungchompu, Pongsatorn Gunun, Nirawan Gunun, Suban Foiklang, Anusorn Thinowong, Yaungyote Jindatajak, Anuwat Lapmee, Thitima Norrapoke

**Affiliations:** 1 Department of Animal Production Technology and Fisheries, Faculty of Agricultural Technology, King Mongkut’s Institute of Technology Ladkrabang, Bangkok 10520, Thailand; 2 Tropical Feed Resources Research and Development Center (TROFREC), Department of Animal Science, Faculty of Agriculture, Khon Kaen University, Khon Kaen 40002, Thailand; 3 Agricultural Unit, Department of Education, National Institute of Education, Phnom Penh 12207, Cambodia; 4 Department of Animal Science, Faculty of Natural Resources, Rajamangala University of Technology Isan, Sakon Nakhon Campus, Sakon Nakhon 47160, Thailand; 5 Department of Animal Science, Faculty of Technology, Udon Thani Rajabhat University, Udon Thani 41000, Thailand; 6 Faculty of Animal Science and Technology, Maejo University, Chiang Mai 50290, Thailand; 7 Sakonnakhon Livestock Research and Breeding Center, Sakon Nakhon 47000, Thailand; 8 Phuphan Sakon Nakhon Dairy Cooperative Ltd., Sakon Nakhon 47000, Thailand; 9 Department of Animal Production Technology, Faculty of Agricultural Technology, Kalasin University, Kalasin 46000, Thailand

**Keywords:** microbial fermented liquid, nutrient digestibility, lactating dairy cows, milk production efficiency, *Saccharomyces cerevisiae*

## Abstract

**Simple Summary:**

The supplementation of direct-feed microorganisms (DFM) that are commonly used in ruminants. The main types of DFM used in ruminant studies include yeast, lactic acid bacteria (LAB), etc. However, the interaction and subsequent results of mixed microbes such as yeast, lactic acid bacteria, and other microbes in ruminants have not been studied. The purpose of this experiment was to examine the effects of microbial fermented liquid (MFL) supplementation on feed intake, nutrient digestibility, milk yield, and milk composition in early lactating dairy cows. The results showed that supplementation could improve feed intake, nutrient digestibility, milk yield, and milk composition in early lactating dairy cows.

**Abstract:**

The purpose of this experiment was to examine the effects of MFL supplementation on feed intake, nutrient digestibility, milk yield, and milk composition in early lactating dairy cows. Twelve, Thai crossbred Holstein Friesian cows in early lactation, 500 ± 30 kg of body weight (BW), were randomly assigned according to a completely randomized design (CRD). MFL supplementation levels of 0, 100, 200, and 300 mL/d were used as treatments. Experimental animals were fed a total mixed ration (TMR) with a roughage to concentrate ratio (R:C ratio) of 40:60, which contains 12% crude protein (CP) and 70% total digestible nutrient (TDN). Rice straw was a roughage source. MFL supplementation levels had no effect (*p* > 0.05) on body weight change and dry matter intake (DMI) expressed as %BW, whereas DMI expressed as metabolic body weight (BW^0.75^) was linearly (*p* < 0.05) increased, with the highest at 200 mL/d in the YFL supplementation group (147.5 g/kg BW^0.75^), whereas feed intake of organic matter (OM), CP, ether extract (EE), neutral detergent fiber (NDF) and acid detergent fiber (ADF) did not significant (*p* > 0.05) difference among treatments. Related to apparent digestibility, MFL levels did not affect (*p* > 0.05) on DM, OM, and EE digestibility, while apparent digestibility of CP, NDF, and ADF were linearly increased (*p* < 0.05) when increasing MFL supplementation levels, and the highest (*p* < 0.05) were the 200 and 300 mL/d FML supplemented groups. BUN at 0 h post feeding did not show a significant difference (*p* > 0.05) between treatments, while at 4 h after feeding, BUN was linearly (*p* < 0.05) increased from 0, 100, 200, and 300 (mL/day) MFL supplementation, the values were 12.9, 13.1, 19.7, and 18.4 mg/dL, respectively and the highest was 200 mL/head/day for the MFL supplemented group. MFL supplementation did not affect (*p* > 0.05) milk fat, lactose, solid not fat (SNF), and specific gravity of milk, while MFL supplementation at 200 mL/day caused a linear increase (*p* < 0.01) in BUN, MUN, milk yield, milk protein, total solids (TS) and 3.5% FCM when supplement levels were increased. In conclusion, MFL supplementation in early lactating dairy cows could improve feed intake, nutrient digestibility, milk yield, and milk composition.

## 1. Introduction

Recently, numerous researchers have tested the use of microorganisms in ruminants’ feed, either directly or as a supplement. Ruminant direct-fed microorganisms (DFM) that are commonly used. The main types of DFM used in ruminants studies include [1] rumen-derived bacteria that can utilize lactic acid (LUB), such as *Megasphaera elsdenii*, *Propionibacterium freudenreichii*, and *Selenomonas ruminantium*, they are modulation of lactic acid metabolism and by promoting the growth of fiber-degrading bacteria or that can convert starch into end products other than lactic acid such as *Prevotella bryantii* 25A, [2] lactic-acid producing bacteria (LAB) of intestinal origin such as bifidobacteria, lactobacilli, and enterococci; They are hypothesized to stimulate the increase of LUB by administering lactic acid as a substrate, thereby improving ruminal pH [1,3] active yeast such as *Saccharomyces cerevisiae* [2]. Active yeast potentially scavenges traces of dissolved oxygen within the rumen, thereby creating an optimal anaerobic growth condition for fibrolytic microorganisms [3]. By limiting the buildup of lactic acid in the rumen, it was also proposed that live yeast would promote optimal growth conditions for bacteria [4].

Polyorach et al. [5] reported that yeast fermented liquid (YFL) consisted of 29.0%DM, 91.7% OM, 8.3% ash, 15.0% CP and 3.2% EE, and that supplementation of YFL with coconut oil in diets that include cassava hay could improve rumen ecology by lowering protozoal populations and increasing bacterial and fungal populations, which improved CP and ADF digestibility in beef cattle. However, the interaction and subsequent results of mixed microbes such as yeast, lactic acid bacteria, and other microbes in ruminants have not been studied.

Recently, Polyorach et al. [6] found that yeast (*Saccharomyces cerevisiae*) and lactic-acid producing bacteria co-cultured in raw milk for 72 h which was the optimal cultivation time for yeast (6.90 log cells/mL) and LAB (9.67 log cells/mL). Moreover, Nampukdee et al. [7] investigated the application of MFL, which was made from effective microorganisms (EM), a liquid mixed culture consisting of lactic acid bacteria, photosynthesis bacteria, and yeast supplementation in concentrate, by using the in vitro gas production technique. It was discovered that 20% MFL supplementation in concentrate improved the kinetics of gas production and increased in vitro digestibility [7]. However, the use of MFL in in vivo trials has not been studied.

It was hypothesized that MFL supplementation could enhance nutrient digestibility, feed intake, and milk production in lactating dairy cows fed a total mixed ration. Therefore, this study aims to examine the effects of MFL supplementation on feed intake and nutrient digestibility in early lactating dairy cows.

## 2. Materials and Methods

The Animal Ethics Committee, in accordance with the Animal Care and Use Committee, King Mongkut’s Institute of Technology Ladkrabang (Approval no. ACUC-KMITL-RES/2023/001) approved all of the experimental animals and methods used in this research.

### 2.1. Microbial Fermented Liquid (MFL) Preparation

The MFL was prepared using the method described by Nampukdee et al. [7]. Effective microorganisms (EM) were obtained from the manufacturer (Bionova Hygiene GmbH, Stans, Switzerland), with each mL containing 1.3 × 10^7^ lactic acid bacteria, 3.3 × 10^4^ photosynthetic bacteria, and 1.3 × 10^4^ yeasts. EM were added to a flask along with 20 g of cane sugar and 100 milliliters of distilled water. The mixture was thoroughly stirred and kept at room temperature (22–33 °C) for one hour. Molasses was weighed at 48 g and dissolved in 100 mL of distilled water, followed by the addition of 48 g of urea to create a liquid culture medium (B). After mixing (A) and (B) in a 1:1 ratio and incubating them for 48 h at room temperature, they can be used in this experiment.

### 2.2. Animals, Diets, and Experimental Design

Sakonnakhon Livestock Research and Breeding Center, Na Kham Road, Phang Khwang Subdistrict, Mueang Sakon Nakhon District, Sakon Nakhon, Thailand, was the location of the experiment. Prior to the commencement of the experiment, cows were of the same age and duration of lactation. Twelve early-lactation crossbred dairy cows (75% HF, 25% Thai native breed) were assigned to 4 levels of MFL supplementation with 3 replications in a completely randomized design (CRD). Control (no supplementation; T1), 100 g/head/day MFL supplementation (T2), 200 g/head/day MFL supplementation (T3), and 300 g/head/day MFL supplementation (T4), respectively, were the treatments. The total mixed ration (TMR) was composed of 40% rice straw and 60% concentrate mixtures (Table 1). The MFL was added to the TMR diets twice a day, at 7.30 a.m. and 4.30 p.m. The microbial fermented liquid was divided into two feedings. Each cow was housed in an individual pen with access to fresh water and mineral blocks. The experiment lasted 30 days, with 23 days dedicated to assessing treatment adaptability and feed intake and the remaining 7 days dedicated to sample collection.

### 2.3. Data Collection and Sampling Procedures

At the start and finish of the experiment, the BW of each animal was determined. During the final week of the experiment, daily samples of feed, refusal, and feces were collected and divided into two equal groups. Half of the samples were analyzed daily for their DM content, while the remaining half were grouped by cow and frozen at −20 °C for chemical analysis. Spot sampling was used to collect the feces, which had a total fresh weight of 50 g/kg. Feed, refusal, and fecal samples were defrosted and oven-dried at 60 °C for 72 h in order to determine the chemical composition. The feed, refusal, and feces samples were then ground through a 1-mm screen and analyzed for dry matter (DM), ash, CP [8], NDF, and ADF [9]. Acid insoluble ash (AIA) was used to evaluate nutrient digestibility [10]. On the final day of the trial, 10 mL of blood was sampled from the jugular vein of each cow at 0 and 4 h after feeding. Each blood sample was stored in EDTA-containing tubes for BUN measurements [11].

Daily milk yield was observed, and milk samples were obtained in the morning (05.00 h) and afternoon (16.00 h) milking times and kept at 4 °C. Measurements of milk were tested for fat, protein, lactose, total solids, and solids-not-fat using infrared methods. The FCM was determined using the following formula: 3.5% FCM = 0.35 milk yield (kg) + 15 fat yield (kg).

### 2.4. Statistical Analysis

The data for a completely randomized design were analyzed using ANOVA in SAS software [12] and the following test model: Y_ij_ = µ + t_i_ + ε_ij_ where Yijk is the observation, μ is the overall mean, t_i_ is effects of treatments 1–4, ε_ij_ is the residual effect. Using Duncan’s new multiple range test, the treatment means were statistically compared [13]. Orthogonal polynomials were used to compare treatment trends. At *p* < 0.05, significant treatment means were acceptable.

## 3. Results

### 3.1. Chemical Composition of Feeds

Cassava chips were the primary energy source, while rice straw was used as the primary source of roughage. The TMR diets were carried out with 12% CP, which satisfied nutrient requirements for dairy cows to produce milk at a rate of 12–15 kg/day. In order to ensure that the animals ingested all of the MFL (20.6% CP), it was top-added to TMR diets at each feeding time.

### 3.2. Changes in Body Weight, Intake and Apparent Digestibility

Effects of MFL supplementation levels on changes in body weight, dry matter intake (DMI), and nutrient intake are shown in Table 2. Animals supplemented with MFL at various levels did not change their final BW or nutrient intakes. However, compared to the group that did not receive MFL (*p* < 0.05), dry matter intake expressed as g/kg BW0^.^75 improved linearly with MFL supplementation. The addition of 200 mL of MFL to the TMR diet significantly improved the digestion of CP, NDF, and ADF (*p* < 0.05).

### 3.3. Blood Urea Nitrogen (BUN)

The effects of MFL supplementation levels on blood urea nitrogen (BUN) are presented in Table 3. BUN at 0 h post-feeding showed no significant difference (*p* > 0.05) between treatments, while at 4 h after feeding, BUN was linearly (*p* < 0.05) increased from 0, 100, 200, and 300 (mL/head/day) MFL supplementation, the values were 12.9, 13.1, 19.7, and 18.4 mg/dL, respectively, and the highest was 200 mL/head/day MFL supplementation.

### 3.4. Milk Yield and Milk Composition

The effects of MFL supplementation on milk yield and milk composition of lactating dairy cows (Table 4). It was found that milk yield was linearly increased (*p* < 0.01) and 3.5% FCM was quadratically increased (*p* < 0.05) when increasing MFL supplement levels, with the highest (*p* < 0.05) at 200 mL supplementation group. Milk protein, total solids, and milk urea-nitrogen levels significantly increased with the addition of MFL at 200 g/d, but milk fat and lactose levels remained unchanged (*p* > 0.05).

## 4. Discussion

Microbial fermented liquid (MFL), a liquid mixed culture made up of yeast, bacteria that participate in photosynthesis, and lactic acid bacteria, should help ruminants better digest their feed. The current findings showed that the apparent digestibility of CP, NDF, and ADF appeared to increase linearly with increasing MFL supplementation. This may be because MFL contains a variety of microorganisms, including yeast and lactic acid bacteria (LAB), which are crucial for boosting the population of rumen microbial digestion activity [14,15]. Polyorach et al. [5] reported that yeast fermented liquid (YFL) could improve bacterial and fungal populations, which improved CP and ADF digestibility in beef cattle.

Furthermore, yeast offers growth-promoting substrates for microbial growth in dairy cows, such as organic acids, amino acids, peptides, and vitamins [16,17]. Moreover, yeast provides an anaerobic condition in the rumen, which is a good environment for rumen microbes, especially cellulolytic bacteria, and increases the binding affinity of anaerobic microbes to feed particles [3,18,19]. Also, yeast cultures encouraged the growth of *Fibrobacter succinogenes* S85, *Ruminococcus albus*, *Butyrivibrio fibrisolvens*, and *Ruminococcus albus* [20,21]. Dry matter intake (DMI), total milk yield, ruminal pH, total volatile fatty acid (VFA) concentrations, and OM digestibility of ruminants are supported by *Saccharomyces cerevisiae* supplementation [18,22]. This is related to what Poppy et al. [23] reported: improved DMI, total milk, milk composition, and milk yield by *Saccharomyces cerevisiae* supplementation.

According to Ferraretto et al. [24], yeast supplementation can increase digestibility, the addition of live *Saccharomyces cerevisiae* increased DM digestibility (4.3 vs. 2.4%) and OM digestibility (3.9 vs. 2.4%). The addition of 4 g/d of yeast increased the digestibility of NDF by 7.5%. Erasmus et al. [25] examined the effect of providing a yeast culture (10 g/d of Yea-Sacc) to lactating Holstein cows fed a high concentrate diet. They found that CP and ADF digestibility increased in comparison to the negative control. Ultimately, an increase in the digestibility of feed is essential for animal production and performance because it increases the passage rate and, consequently, DMI in ruminants.

Polyorach et al. [5] reported that yeast fermented liquid (YFL) contained 29.0% DM, 91.7% OM, 8.3% ash, 15.0% CP, and 3.2% EE and that supplementing with coconut oil with cassava hay diets could improve rumen ecology by decreasing protozoal populations and increasing bacterial and protozoal populations, improving CP and ADF digestibility compared to diets containing soybean meal. Determining the application of yeast in ruminants necessitates further examination of the confounding variables (i.e., yeast type, dose, and processing) that influence its efficacy.

In Nampukdee et al. [7] comparison between supplementation of yeast and MFL in a concentrate diet by using an in vitro gas production technique, it was found that the in vitro dry matter degradability (IVDMD) of the 20% yeast supplemented group was the highest (*p* < 0.01) (79.52%), and the 0% yeast supplemented group was the lowest (68.78%). Furthermore, the in vitro organic matter degradability (IVOMD) of the 20% yeast supplemented group was the highest (*p* < 0.01) (96.75%), and the 0% yeast supplemented group was the lowest (92.96%).

The primary components of MFL are lactic acid-producing bacteria (LAB) (including *Lactobacillus* spp., *Streptococcus* spp., *Pediococcus* spp., and *Enterococcus* spp.), yeast, and other microorganisms [7]. MFL also helps in the manipulation and efficiency of the rumen in ruminants. MFL is capable of balancing the intestinal microflora of animals, which increases nutrient absorption and reduces methane production. Additionally, lactic acid bacteria in MFL support fermentation and cellulose and lignin degradation [18].

McAllister et al. [2] demonstrated that LAB are desirable as directly fed microorganisms (DFM) because they are amenable to industrial culture, are environmentally reliable, and possess a variety of mechanisms by which they are able to modify or affect microbial communities.

The mechanism of LAB is to change the intracellular pH of bacterial competitors and product antimicrobial peptides (bacteriocins) [26]. LAB can inhibit Salmonella activity in vitro by producing hydrogen peroxide in an aerobic condition [27]. It is unknown, however, what function these antimicrobials play in the intestinal tract, where oxygen levels are low. To achieve a multifactorial effect, many commercial DFM products contain LAB in addition to other bacteria or yeast. Although this strategy makes production sense, it makes it extremely difficult to attribute performance or pathogen exclusion responses to a particular microbial component within the DFM. Additionally, it is not possible to rule out the possibility that mixed DFM may have interactive effects on performance characteristics.

Polyorach et al. [6] discovered that 72 h post-cultivation was the optimal cultivation time for yeast (6.90 log cells/mL) and LAB (9.67 log cells/mL) populations co-cultured in fermented milk. It has been hypothesized that interactions between LAB and yeasts could affect the product’s characteristics and quality due to their frequent co-occurrence [26,28]. Moreover, yeast and lactic acid bacteria co-cultured in fermented milk could improve the nutritional value of soybean meal in terms of CP, EE, NDF, and ADF from 46.8, 3.2, 12.8, and 10.2, respectively, to 58.9, 6.2, 11.3, and 6.5, respectively. Soybean meal fermented milk (SBMFM) products are a high-quality source of protein that could improve degradability in the rumen, especially when added at 5% of the total concentrate substrate by using the nylon bag technique [6].

Blood urea nitrogen (BUN) was linearly increased by MFL supplementation. The BUN concentrations corresponded to the digestibility of protein nutrients and were related to the concentration of ammonia-nitrogen (NH_3_-N) in the rumen [29,30]. This explains that the increase in BUN is a result of the animal’s diet containing high levels of protein or non-protein nitrogenous compounds [31,32]. When it enters the rumen, microorganisms there, especially the proteolytic bacteria group, can digest more NH_3_-N [33], which is then absorbed through the rumen wall into the bloodstream and transported into the liver to be converted into more urea, resulting in increased BUN levels [34,35]. In addition, an increase in BUN resulted in an increase in the concentration of MUN [29,36].

Milk urea nitrogen (MUN) of 200 mL/head/day from MFL supplementation was the highest (15.4 mg/100 mL). MUN concentrations under the current study were similar to those reported by Jonker et al. [37], who reported that the optimum value of MUN should be between 10 and 16 mg/100 mL. The MUN concentration indicates adequate protein levels in the diet and a balance of protein and energy in the feed [38,39].

Furthermore, the increase in milk protein percentage was consistent with higher digestibility of protein nutrients in feed intake and an increasing BUN concentration. Polyorach et al. [40] reported that supplementation of mangosteen peel powder at 300 g/hd/d with yeast-fermented cassava chips (YEFFCAP) as a protein source could increase the milk protein percentage in dairy cows. Similar to the report by Dias et al. [41], yeast culture was studied in dairy cows fed a high starch diet. It was found that the percentage of protein intake was increased (1.18 kg/day). The increased milk protein under the current study might be due to improved rumen fermentation, which affects protein degradation and amino acid content [42]. In addition, yeast, when decomposed, is also a nutrient for bacteria in the rumen. In particular, the proteolytic bacteria can produce amino acids and ammonia that can be absorbed by dairy cows through the rumen wall and absorbed through the intestine for use in increasing the percentage of protein in milk [43].

## 5. Conclusions

Based on this study, it could be concluded that MFL supplementation levels in early lactating dairy cows did not affect DM, OM, and EE digestibility, milk fat, milk lactose, solid not fat (SNF), or the specific gravity of milk. However, DMI, digestibility of CP, NDF, and ADF, BUN, MUN, milk yield, milk protein, total solids (TS), 3.5% FCM were improved when 200 mL/day of MFL was supplemented. Supplementation in early lactation dairy cows may therefore improve feed intake, nutrient digestibility, milk output, and milk composition. Further research should be done on the supplementing impact of MFL on rumen parameters, rumen microorganisms, and higher animal numbers.

## Figures and Tables

**Table 1:** Chemical composition of total mixed ration (TMR) and microbial fermented liquid (MFL).

Item	TMR	MFL	Rice Straw
Ingredient, % of DM			
Rice straw	40.0		
Cassava chip	39.6		
Rice bran	5.0		
Soybean meal	8.5		
Urea	2.0		
Molasses	2.0		
Tallow	2.0		
Salt	0.3		
Sulfur	0.3		
Mineral mixture ^1^	0.3		
Chemical composition			
Dry matter (DM), %	89.6	22.2	90.2
	% of dry matter		
Organic matter (OM)	90.7	98.9	83.0
Crude protein (CP)	12.3	20.6	2.7
Ether extract (EE)	3.5	1.2	2.0
Neutral detergent fiber (NDF)	56.4	-	80.4
Acid detergent fiber (ADF)	28.3	-	54.0

^1^ Minerals and vitamins (each kg contained): IU: vit. A 10,000,000, vit. E 70,000, vit. D 1,600,000; g: Fe 50, Zn 40, Mn 40, Co 0.1, Cu 10, Se 0.1, I 0.5.

**Table 2:** Effects of MFL supplementation levels on body weight change, dry matter intake (DMI) and nutrient intake.

Item	Supplement Levels (mL/hd/d) ^1^				SEM	Contrasts ^2^	
	0	100	200	300		L	Q
Body weight							
Initial body weight (kg)	512.0	505.3	505.7	517.3	19.32	0.22	0.66
Final body weight (kg)	531.0	526.3	529.0	539.5	20.35	0.32	0.45
Dry matter intake (DMI)							
Kg/d	13.3	15.3	15.7	15.5	0.34	0.27	0.22
%BW	2.6	3.0	3.1	3.1	0.09	0.78	0.77
g/kg BW ^0.75^	123.9 ^b^	143.8 ^a^	147.5 ^a^	145.5 ^a^	3.33	0.03	0.43
Nutrient intake (kg/hd/d)							
Organic matter (OM)	12.1	13.9	14.3	14.1	0.32	0.66	0.83
Crude protein (CP)	1.6	1.8	1.9	1.9	0.04	0.54	0.69
Ether extract (EE)	0.4	0.5	0.6	0.5	0.01	0.45	0.92
Neutral detergent fiber (NDF)	7.5	8.6	8.9	8.7	0.20	0.67	0.18
Acid detergent fiber (ADF)	3.8	4.3	4.5	4.4	0.10	0.97	0.65
Apparent digestibility (%)							
Dry matter (DM)	65.0	64.6	68.4	66.9	2.22	0.23	0.55
Organic matter (OM)	65.4	67.1	70.7	68.45	1.65	0.22	0.34
Crude protein (CP)	50.5 ^b^	53.2 ^b^	69.1 ^a^	67.3 ^a^	1.07	0.01	0.43
Ether extract (EE)	66.7	71.4	73.2	72.6	0.96	0.45	0.89
Neutral detergent fiber (NDF)	49.6 ^b^	55.1 ^ab^	63.2 ^a^	61.9 ^a^	1.52	0.02	0.65
Acid detergent fiber (ADF)	49.1 ^b^	55.0 ^ab^	64.3 ^a^	59.7 ^ab^	1.69	0.05	0.44

^a,b^ Means in the same row with different superscripts, SEM = standard error of the means, ^1^ Levels of microbial fermented liquid supplementation, ^2^ L = Linear, Q = Quadratic.

**Table 3:** Effects of MFL supplementation levels on blood urea nitrogen (BUN).

Item	Supplement Levels (mL/hd/d)				SEM	Contrasts	
	0	100	200	300		L	Q
BUN, mg/dL							
0 h post feeding	11.1	11.7	12.4	12.7	0.29	0.44	0.98
4 h post feeding	12.9 ^b^	13.1 ^b^	19.7 ^a^	18.4 ^a^	0.25	0.01	0.78
Average	12.1 ^b^	12.4 ^b^	16.1 ^a^	15.6 ^a^	0.08	0.01	0.04

^a,b^ Means in the same row with different superscripts.

**Table 4:** Effects of MFL supplementation levels on milk yield, and milk composition in lactating dairy cows.

Item		Supplement Levels (mL/hd/d)				SEM	Contrasts	
		0	100	200	300		L	Q
Production								
Milk yield (kg/hd/d)		12.6 ^b^	14.0 ^ab^	15.4 ^a^	14.8 ^a^	0.22	0.01	0.23
3.5% Fat corrected-milk (kg/hd/d)		12.7 ^c^	14.9 ^b^	17.7 ^a^	16.3 ^ab^	0.32	0.01	0.04
Milk composition, %								
Fat		3.4	3.9	4.5	4.2	0.17	0.23	0.15
Protein		3.2 ^b^	3.7 ^ab^	4.0 ^a^	3.7 ^ab^	0.09	0.03	0.23
Lactose		4.2	4.4	4.8	4.6	0.15	0.33	0.27
Solid not fat (SNF)		8.1	8.5	8.8	8.6	0.12	0.44	0.34
Total solid (TS)		10.8 ^b^	12.2 ^ab^	13.0 ^a^	12.6 ^a^	0.23	0.04	0.66
Specific gravity		1.03	1.03	1.03	1.02	0.0003	0.54	0.45
Milk urea-nitrogen (MUN), mg/100 mL								
		11.9 ^b^	12.2 ^bc^	15.4 ^a^	14.4 ^ab^	0.39	0.03	0.43

^a,b,c^ Means in the same row with different superscripts.

## Data Availability

Not applicable.
